# Rat Hair Follicle Stem Cell-Derived Exosomes: Isolation, Characterization and Comparative Analysis of Their In Vitro Wound Healing Potential

**DOI:** 10.3390/ijms26115081

**Published:** 2025-05-25

**Authors:** Patrícia Sousa, Bruna Lopes, Ana Catarina Sousa, Alícia de Sousa Moreira, Alexandra Rêma, Rui Alvites, Stefano Geuna, Nuno Alves, Ana Colette Maurício

**Affiliations:** 1 Departamento de Clínicas Veterinárias, Instituto de Ciências Biomédicas de Abel Salazar (ICBAS), Universidade do Porto (UP), Rua de Jorge Viterbo Ferreira, No. 228, 4050-313 Porto, Portugal; pfrfs_10@hotmail.com (P.S.); brunisabel95@gmail.com (B.L.); anacatarinasoaressousa@hotmail.com (A.C.S.); alicia.moreira.1998@gmail.com (A.d.S.M.); alexandra.rema@gmail.com (A.R.); ruialvites@hotmail.com (R.A.); 2 Centro de Estudos de Ciência Animal (CECA), Instituto de Ciências, Tecnologias e Agroambiente da Universidade do Porto (ICETA), Rua D. Manuel II, Apartado 55142, 4051-401 Porto, Portugal; 3 Associate Laboratory for Animal and Veterinary Science (AL4AnimalS), 1300-477 Lisboa, Portugal; 4 Instituto Universitário de Ciências da Saúde (IUCS), Instituto Universitário de Ciências da Saúde (CESPU), Avenida Central de Gandra 1317, 4585-116 Paredes, Portugal; 5 Centre for Rapid and Sustainable Product Development, Polytechnic of Leiria, 2430-028 Marinha Grande, Portugal; stefano.geuna@unito.it; 6 Department of Clinical and Biological Sciences, Cavalieri Ottolenghi Neuroscience Institute, University of Turin, Ospedale San Luigi, 10043 Turin, Italy; nuno.alves@ipleiria.pt

**Keywords:** exosomes characterization, exosomes isolation, extracellular vesicles, hair follicle stem cells, regenerative medicine, wound healing

## Abstract

Stem cell-derived secretome and exosomes present a promising cell-free strategy for tissue repair and wound healing. This study aimed to isolate and characterize, for the first time, exosomes derived from rat hair follicle stem cells (rHFSCs) and to evaluate their wound-healing potential alongside rHFSC secretome. Exosomes were isolated via ultracentrifugation and characterized using Reverse Transcriptase Polymerase Chain Reaction (RT-PCR), biomarker profiling and protein quantification. Scanning electron microscopy (SEM) with energy-dispersive X-ray spectroscopy (EDS) confirmed their spherical morphology, diameter and elemental composition. Protein quantification showed higher protein content in the secretome than in exosomes. RT-PCR and biomarker profiling highlighted the therapeutic relevance of the exosomal cargo compared to parent rHFSCs. Functional analysis of 30 wound-healing biomolecules validated their pro-regenerative potential. Cytocompatibility was confirmed via the PrestoBlue™ viability assay, while scratch assays demonstrated significant wound closure in the treated groups, both with and without mitomycin C. These findings highlight the potential of rHFSC-derived exosomes and secretome as innovative, cell-free therapeutic agents for cutaneous regeneration. This study advances our understanding of their role in wound healing and underscores their broader applicability in regenerative medicine.

## 1. Introduction

The skin is the largest organ of the body, serving as a protective barrier against environmental damage, pathogens and dehydration while regulating body temperature and enabling sensory perception. Composed of three main layers—epidermis, dermis and hypodermis—it is a dynamic tissue that undergoes continuous renewal. The epidermis primarily consists of keratinocytes and serves as the primary defense. The dermis provides structural support through collagen and elastin fibers, while the hypodermis contains adipose tissue for insulation and cushioning. The skin’s remarkable capacity for repair is driven by cellular components such as keratinocytes, fibroblasts and immune cells, working alongside molecular signals like growth factors and cytokines. However, in certain cases, such as severe burns, chronic wounds or diabetic ulcers, the skin’s natural regenerative ability is impaired, needing alternative therapeutic interventions [1,2,3].

Skin lesions that fail to regenerate properly, such as chronic wounds or extensive burns, pose significant clinical challenges. These wounds may persist due to disrupted healing mechanisms, including impaired angiogenesis, chronic inflammation or deficient extracellular matrix (ECM) remodeling. Traditional treatments like surgical debridement, skin grafting and synthetic dressings often fall short in promoting full functional and aesthetic restoration, especially in cases of large-scale tissue damage [4,5,6].

To address these limitations, alternative approaches are being explored, focusing on enhancing the skin’s natural healing potential. Advances in biomaterials, cell-based therapies and biological products aim to create a conducive environment for tissue regeneration. Bioengineered scaffolds, platelet-rich plasma and autologous skin cell transplants have shown potential in improving wound outcomes [7,8]. However, these methods still have limitations in terms of availability, cost and effectiveness for large or complex wounds, driving interest in newer, innovative therapies [6,9,10].

Emerging therapies such as secretome and exosomes derived from stem cells offer promising solutions for wound healing. Secretome offers significant advantages due to its rich content of bioactive molecules such as growth factors, cytokines and extracellular vesicles (EVs).

When compared to cell-based therapies, secretome reduces the risks related to immune rejection and tumor formation, making them a safer option. Moreover, secretome helps to modulate inflammation, accelerate angiogenesis and stimulate ECM remodeling, all of which contribute to faster wound closure and improved tissue quality [11,12,13,14,15].

Several studies demonstrated that stem cells’ secretome from different sources significantly enhances wound closure rates, while reducing neutrophil and macrophage infiltration, underscoring its anti-inflammatory properties [15,16,17,18].

The EVs are cell-derived structures that facilitate communication and regulate physiological processes such as tissue repair [19,20,21]. A key advantage of EVs is their ability to cross biological barriers, including the blood–brain barrier and cell membranes. Among them, there is a specialized subtype identified as the exosomes. These molecules play a crucial role in promoting tissue regeneration by enhancing cell proliferation, migration and differentiation, which are vital for effective wound repair. Exosomes are nano-sized vesicles ranging from 30 to 200 nm, enclosed by a lipid bilayer that can encapsulate both hydrophobic and hydrophilic drugs. Their surface is rich in immune regulatory molecules, membrane proteins and trafficking molecules, enabling selective attachment to target sites and enhancing their role in biomolecule delivery and intercellular communication [22,23]. These vesicles transport several biological components, including mRNA, nucleic acids, protein chaperones, lipids and cytoplasmic components, allowing them to modulate the physiological or pathological functions of recipient cells [24]. Exosomes deliver their cargo through multiple mechanisms, including ligand–receptor interactions that activate signaling pathways, as well as pinocytosis, phagocytosis and direct fusion with the plasma membrane. These nanoscale vesicles are particularly potent due to their diverse cargo of growth factors, cytokines and microRNAs, which play an active role in cellular processes, such as wound healing. Their small size enhances cellular uptake, while their lipid bilayer protects their molecular cargo, ensuring stability and prolonged circulation time. This targeted and controlled delivery system makes exosomes highly effective in promoting angiogenesis, reducing inflammation and activating fibroblasts to support ECM remodeling. Unlike the broader, unfractionated secretome—which contains a complex and variable mixture of molecules which includes other extracellular vesicles such as microvesicles—exosomes offer a more standardized and reproducible therapeutic approach. Furthermore, being acellular, exosomes pose a lower risk of immune rejection and off-target effects, making them a promising, minimally invasive option for treating chronic or non-healing wounds [25,26,27,28,29,30,31,32,33].

Among the various sources of stem cell-derived exosomes, HFSCs stand out due to their high multipotency and accessibility via minimally invasive procedures. Exosomes derived from rHFSCs have shown unique regenerative properties, especially in skin and hair follicle repair, likely owing to their ectodermal origin, which may confer advantages in dermatological applications [2,34]. Compared to other cells, such as mesenchymal stem cells (MSCs) or adipose-derived stem cells, rHFSC-derived exosomes may possess a distinct miRNA and protein cargo, making them particularly effective in modulating epithelial and dermal cell interactions [35,36]. Despite these promising attributes, rHFSC-derived exosomes remain relatively underexplored in the context of wound healing.

Studies using mouse and rat models of wound healing demonstrated that exosome treatment significantly improved wound healing, particularly by promoting angiogenesis. Exosomes accelerated wound closure, enhanced vascularity and fostered tissue regeneration, with most studies reporting notable therapeutic benefits [1,19,37,38,39,40,41]. These effects suggest that exosomes can actively modulate key biological pathways involved in skin healing, addressing common challenges such as poor vascularization and delayed tissue repair. Although current studies are still limited, the consistent positive outcomes observed in preclinical models highlight exosomes as a promising and innovative strategy for treating skin injuries and disorders. Further research is needed to fully understand their mechanisms of action and to translate these findings into clinical practice, but the existing evidence points to a substantial therapeutic advantage that could revolutionize approaches to skin regeneration.

This study is the first to isolate and characterize exosomes derived from rat hair follicle stem cells (rHFSCs) for in vitro assessment of their wound healing potential, while comparing it to the plain secretome. It underscores the potential of rHFSC-derived components as innovative, cell-free therapeutic agents, offering promising alternatives for enhancing skin regeneration and accelerating wound healing. Additionally, it provides a valuable preclinical foundation for future in vivo studies.

## 2. Results

### 2.1. rHFSC-Derived Exosomes Analysis and Comparison to Secretome

**Figure 1:**
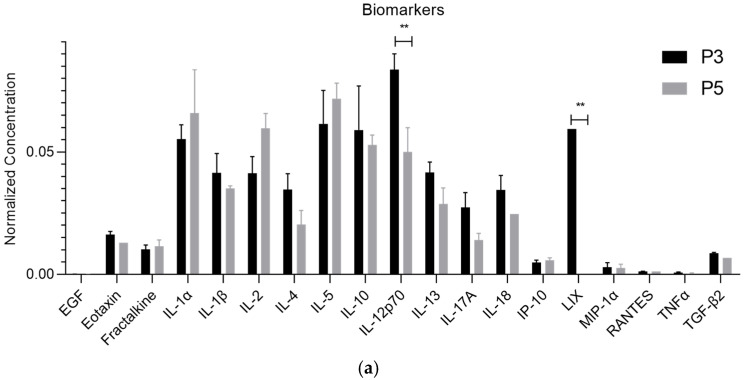

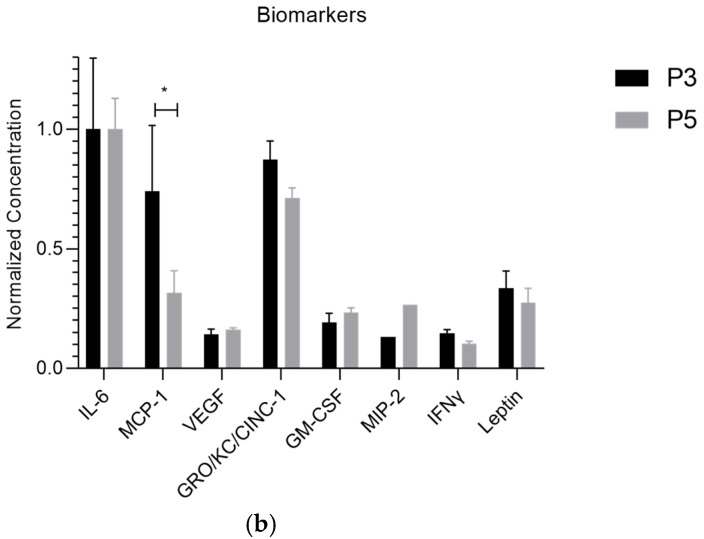
Normalized concentration of each biomolecule present in rHFSC-derived exosomes (mean ± SEM)—(**a**) Biomolecules with lower normalized concentrations; (**b**) biomolecules with higher normalized concentrations. The division into two panels allows better visualization due to the wide variation in concentration levels. Statistical significance is indicated by the symbols *p* < 0.05 (*) and *p* < 0.01 (**).

**Figure 2:**
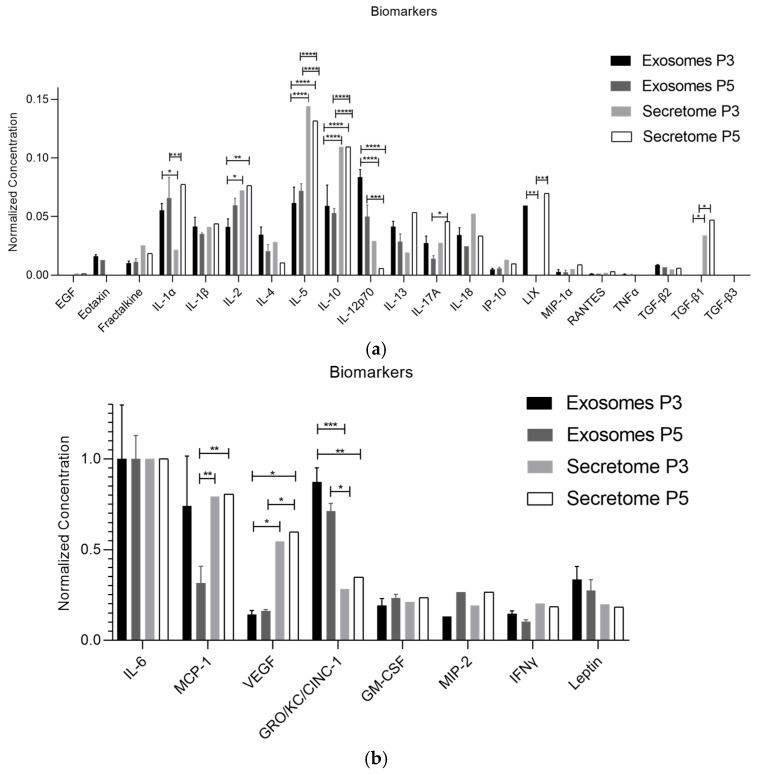
Comparison of normalized concentrations of each biomolecule present in rHFSC-derived exosomes and secretome (mean ± SEM)—(**a**) biomolecules with lower normalized concentrations; (**b**) biomolecules with higher normalized concentrations. Panels were separated to enhance visualization due to the broad range of concentration values. The significance of the results is indicated by the symbols (*), with (*) corresponding to 0.01 ≤ *p* < 0.05, (**) to 0.001 ≤ *p* < 0.01, (***) to 0.0001 ≤ *p* < 0.001 and (****) to *p* < 0.0001.

The mean concentration values of each biomarker detected in the exosomes and secretome are presented in Table 1 and Figure 1. Figure 2 and Table 2 provide a comparative analysis of biomarker profiles between the secretome and exosomes across passages P3 and P5. Notably, Figure 1 showcases the molecular characterization of exosomes derived from rHFSCs, a novel aspect of this study that has not been previously reported. While the secretome data have been published earlier, the side-by-side visual comparison in Figure 2 highlights distinct differences in biomolecule composition, offering meaningful insights into the selective packaging mechanisms of exosomes and their potential advantages in regenerative applications compared to other secreted factors [2].

### 2.2. RT-PCR

**Figure 3:**
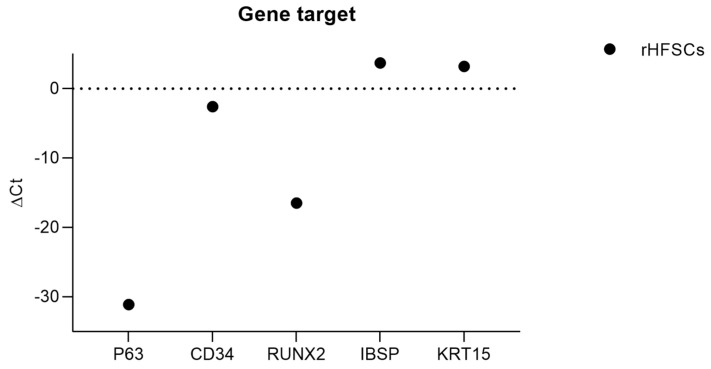
ΔCt values for each gene under study. Higher ΔCt values demonstrate lower expression.

To assess the molecular profile of exosomes derived from rHFSCs, RT-PCR analysis was conducted and compared to the gene expression of the parent cells, using GAPDH as a stable reference gene. Gene expression levels were categorized as high (Ct < 29), moderate (29 ≤ Ct ≤ 35) or low (Ct ≥ 35) based on Ct values, with negative ΔCt values indicating upregulation in exosomes (Table 3 and Figure 3). The detection of p63, a key stemness marker, confirmed the epithelial origin of the exosomes [42,43], while the presence of CD34 and KRT15 suggested that they retained stem-like characteristics from their parent cells [44,45,46]. Additionally, the expression of RUNX2 and IBSP pointed to potential osteogenic activity [47,48]. However, several genes related to differentiation and extracellular matrix composition—such as KRT14, KRT10, KRT19, COL2A1, ITGA6, ACAN, ITGB1, ADIPOQ and AAK1—were not detected [2]. Overall, the results indicate that rHFSC-derived exosomes selectively carry genetic material related to stemness and early differentiation, supporting their potential role in regenerative signaling.

### 2.3. Scanning Electron Microscopy (SEM) with Energy-Dispersive X-Ray Spectroscopy (EDS)

**Figure 4:**
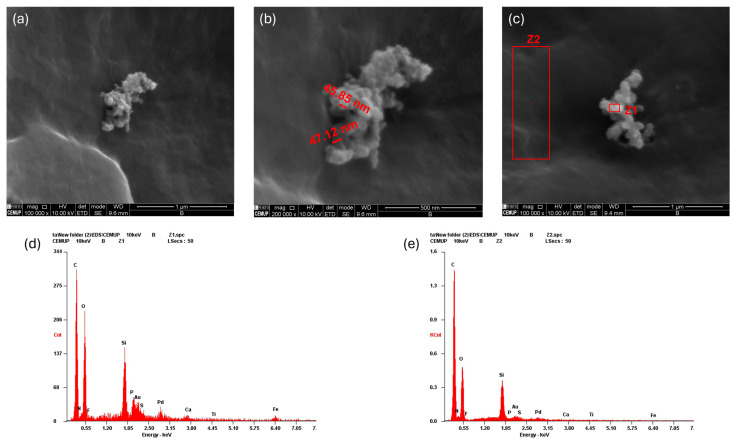
SEM with EDS analysis of rHFSC-derived exosomes: (**a**) SEM image of exosomes; (**b**) measurement of exosome size; (**c**) identification of exosome regions for elemental analysis; (**d**) EDS spectrum of exosome region Z1; and (**e**) EDS spectrum of exosome region Z2.

The SEM with EDS analysis enabled the characterization of the isolated exosomes in terms of their morphology and size—Figure 4.

Figure 4 demonstrated that the exosomes possessed a typical spherical or cup-shaped morphology, consistent with their nanoscale size and uniform distribution. The majority of the vesicles measured between 40 nm and 60 nm in diameter, aligning with the lower end of the expected exosome size range, though occasional larger sizes may occur depending on biological and methodological factors. High-resolution imaging confirmed the structural integrity and surface uniformity of the exosomes. Elemental analysis through EDS in two zones (Z1 and Z2) revealed strong signals for carbon and oxygen, indicating the presence of lipids and proteins that constitute the exosomal membrane. The detection of nitrogen, phosphorus and sulfur further suggests the presence of nucleic acids and proteins within the vesicles. Trace amounts of gold and palladium likely resulted from SEM sample preparation, while elements such as silicon, calcium, titanium and iron may reflect interactions with the substrate. Differences in elemental intensity between Z1 and Z2 point to possible heterogeneity in surface composition or clustering behavior.

### 2.4. Total Protein Quantification

**Figure 5:**
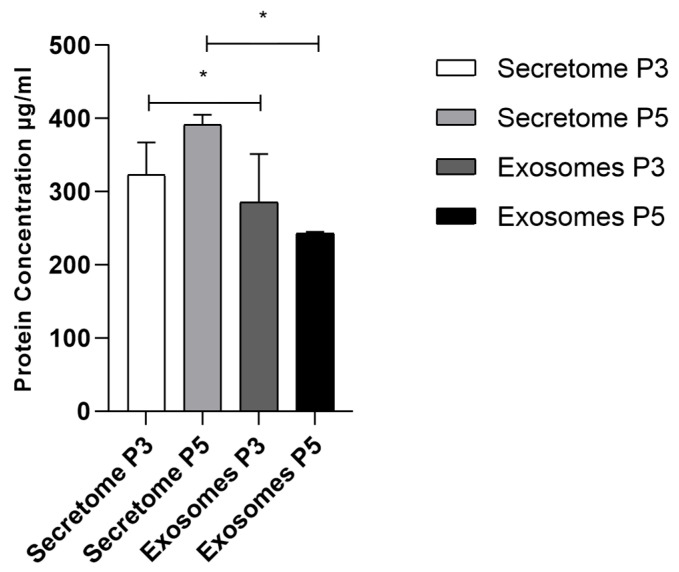
Protein concentration for secretome and exosomes isolated from cells in P3 and P5 (mean ± SEM). The significance of the results is indicated by symbols (*), with (*) corresponding to 0.01 ≤ *p* < 0.05.

Figure 5 and Table 4 show the protein concentration of secretome and exosome isolated from cell samples at P3 and P5. Exosome yield was quantified, and results are expressed as μg of total protein per mL.

The graphic illustrates that the protein concentration in secretome samples is consistently higher than in exosome samples at both passages.

Figure 5 shows that the protein concentration is significantly higher in the secretome than in exosomes at both P3 and P5 passages. Among the exosome samples, those from P3 exhibit a slightly higher protein concentration than those from P5. NanoDrop analysis further supports these findings, revealing greater concentration and purity in P3-derived exosomes. These results suggest that exosomal protein yield and quality may decline with increasing passage number, which could impact their effectiveness in downstream applications.

### 2.5. Prestoblue Assay

**Figure 6:**
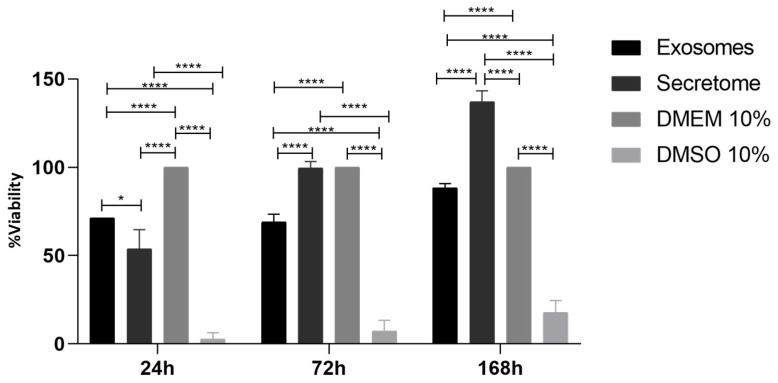
Percentage of cell viability after exosomes and secretome derived from rHFSCs contact with L929 cells up to 168 h. Results presented as mean ± SEM. Results significances are presented through the symbol (*), according to the *p*-value, with (*) corresponding to 0.01 ≤ *p* < 0.05 and (****) to *p* < 0.0001.

**Figure 7:**
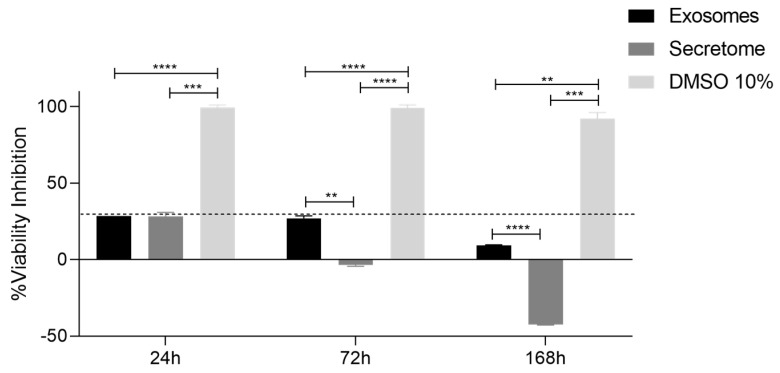
Percentage of cell viability inhibition after the direct contact of L929 cells with exosomes and secretome up to 168 h. Results presented as mean ± SEM. The dashed line represents the percentage of cell viability inhibition above which cytotoxicity is considered, according to ISO 10993-5:2009 [49], Results significances are presented through the symbol (*), according to the *p*-value, with (**) to 0.001 ≤ *p* < 0.01, (***) to 0.0001 ≤ *p* < 0.001 and (****) to *p* < 0.0001.

The cytocompatibility results of L929 cells contact with rHFSC-derived exosomes and rHFSC-derived secretome after 24, 72 and 168 h are presented in Figure 6 and Table 5. The percentage of cell viability inhibition over time is presented in Figure 7 and Table 6.

The viability assay results indicate that both exosomes and secretome are cytocompatible with L929 cells. At 24 h, exosomes and secretome showed similar viability, both significantly higher than the cytotoxic DMSO 10% group, though slightly lower than the DMEM control. By 72 h, secretome-treated cells maintained viability comparable to the DMEM control, while exosome-treated cells showed slightly reduced viability but remained well above the DMSO group. At 168 h, secretome-treated cells exhibited a marked increase in viability, surpassing all other groups, suggesting a potential long-term proliferative effect. Exosome-treated cells retained viability similar to the DMEM group throughout. Importantly, both treatments consistently demonstrated cell viability inhibition below the 30% cytotoxicity threshold set by ISO 10993-5:2009 [49], confirming their cytocompatibility.

### 2.6. Scratch Assay

**Figure 8:**
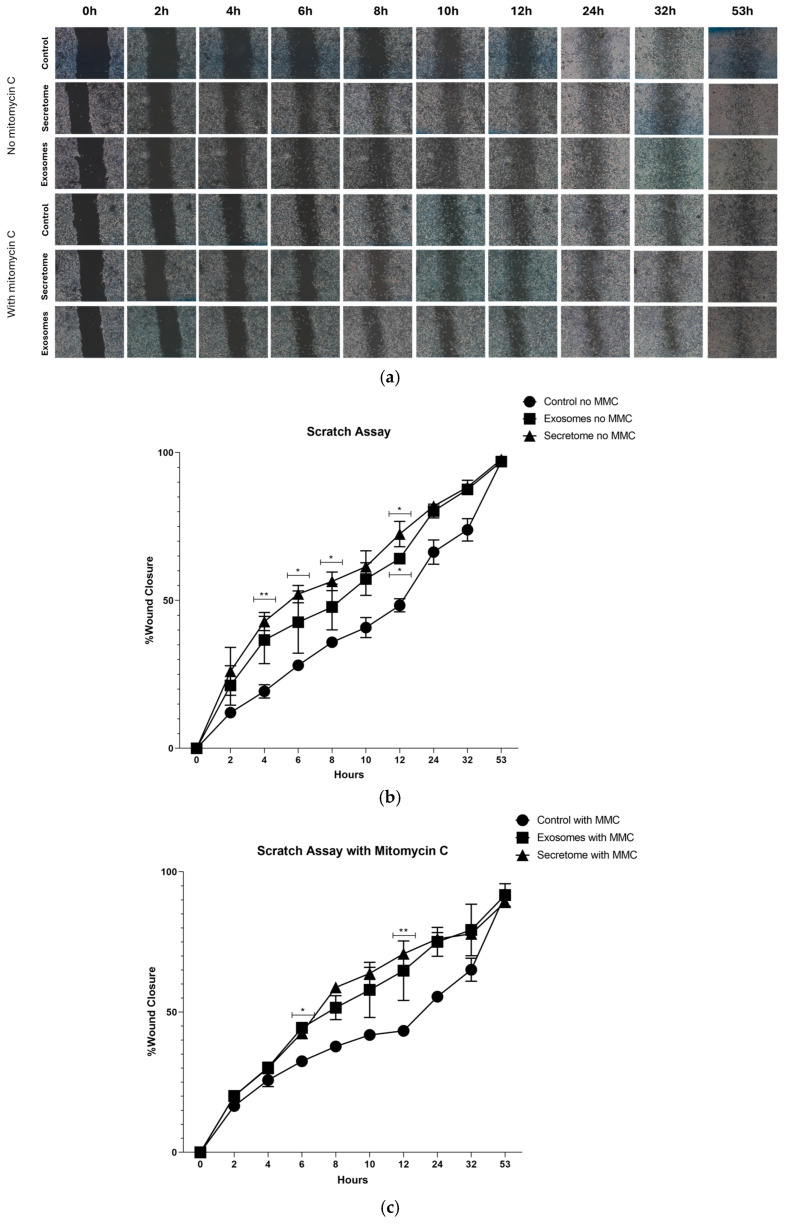
Effects of rHFSC-derived secretome and exosomes on wound healing in L929 cells—(**a**) representative images of wound healing scratch assays at various time points (0, 2, 4, 6, 8, 10, 12, 24, 32 and 53 h) following treatment with rHFSC-derived secretome and exosomes, with and without MMC; (**b**) quantification of wound closure percentage over time (mean ± SEM). Statistical significance is indicated by symbols (*) for 0.01 ≤ *p* < 0.05 and (**) for 0.001 ≤ *p* < 0.01 and (**c**) wound closure percentage over time in L929 cells pretreated with MMC, presented as mean ± SEM. Statistical significance is indicated by symbols (*) for 0.01 ≤ *p* < 0.05 and (**) for 0.001 ≤ *p* < 0.01. MMC = Mitomycin C.

Figure 8 illustrates the wound closure dynamics in the L929 cell line over time, with and without MMC treatment, following exposure to exosomes and secretome derived from rHFSCs. The results indicate that the treatment groups enhance both cell migration and proliferation at earlier time points, as by the end of the assay (53 h), all wounds were completely closed in all groups. Table 7 and Table 8 demonstrate the statistical difference between groups at different timepoints.

## 3. Discussion

The rHFSCs have garnered considerable interest due to their pro-regenerative potential and their role in cell-to-cell communication, making them a promising approach for therapeutic applications. Exosomes, as key components of the rHFSC secretome, play a crucial role in mediating tissue repair and modulating immune responses.

This study aimed to establish a novel methodology for the isolation, characterization and storage of rHFSC-derived exosomes, as well as to evaluate their bioactive cargo and wound-healing properties.

A straightforward and reproducible isolation protocol was developed, ensuring the integrity and purity of the exosome preparations. The isolation process was carried out under aseptic conditions, incorporating ultracentrifugation techniques to achieve a high yield and contamination-free exosome fractions. Detailed methodological descriptions were provided to enhance reproducibility and reliability in future studies. The exosome isolation protocol presented in this study differs from previously established methods due to the specific rHFSC cell line used, which provides a unique microenvironment for exosome secretion.

To characterize the isolated exosomes, several analytical techniques were employed. Specific biomarkers associated with wound healing were analyzed to validate the regenerative potential of rHFSC-derived exosomes. The biomolecules with the highest expression in the analysis—IL-6, MCP-1, VEGF, GRO/KC/CINC-1, GM-CSF, MIP-2, IFN-γ and leptin—play crucial roles in wound healing by coordinating inflammation, angiogenesis and tissue regeneration. IL-6 and IFN-γ drive the inflammatory phase, promoting immune cell recruitment and activation. MCP-1 and MIP-2 facilitate monocyte and neutrophil chemotaxis, aiding in pathogen clearance and tissue remodeling. VEGF is essential for angiogenesis, ensuring sufficient oxygen and nutrient delivery to regenerating tissues. GRO/KC/CINC-1 enhances neutrophil recruitment, while GM-CSF supports macrophage activation and tissue repair. Additionally, leptin contributes to fibroblast proliferation and ECM remodeling. The elevated expression of these biomolecules suggests a highly dynamic wound-healing environment, where inflammation, angiogenesis and tissue remodeling work in synergy to restore tissue integrity [50,51,52]. Beyond these key factors, the analysis revealed variations in several other cytokines and chemokines involved in immune modulation and tissue remodeling. EGF, eotaxin, IL-1α, IL-1β, IL-2, IL-4, IL-5, IL-12p70, IL-17A, IP-10, MIP-1α, RANTES, TNFα and TGF-β2 were detected, albeit at lower levels. EGF plays a crucial role in epithelial cell proliferation and migration, while eotaxin regulates eosinophil recruitment during immune responses. IL-1α and IL-1β contribute to early inflammatory signaling, stimulating immune activation, while IL-2, IL-4 and IL-5 are involved in T-cell differentiation and immune regulation. IL-12p70 is essential for Th1 immune responses, whereas IL-17A modulates neutrophil recruitment and inflammation. IP-10 and MIP-1α are chemokines that enhance immune cell trafficking, while RANTES is involved in leukocyte recruitment. TNFα plays a dual role in both pro-inflammatory responses and tissue repair, whereas TGF-β2 is linked to ECM remodeling and fibrosis regulation [53,54]. An interesting distinction between exosomes and the broader secretome is the absence of TGF-β1 and TGF-β3 in the exosome profile despite their presence in the secretome. This suggests that these growth factors are predominantly secreted in a free or soluble form rather than being packaged into exosomes. Given their crucial roles in wound healing—particularly in fibrosis regulation and ECM remodeling—their absence in exosomes may indicate a preference for direct paracrine signaling rather than exosomal transport. In contrast, the higher expression of LIX in the exosome profile suggests that this chemokine is selectively packaged into exosomes, possibly to enhance immune cell recruitment or modulate the inflammatory response at distant sites. As a potent neutrophil chemoattractant, LIX’s enrichment in exosomes may serve to amplify localized inflammatory signaling or extend its bioavailability compared to its soluble form. Notably, a statistical difference was observed between P3 and P5 exosomes, with LIX, IL-12p70 and MCP-1 being significantly higher in P3 exosomes compared to P5 exosomes. Additionally, when compared to the secretome, MCP-1, VEGF, GRO/KC/CINC-1, IL-1α, IL-2, IL-5, IL-10, IL-12p70, IL-17A, LIX and TGF-β1 showed significant differences. Overall, these findings highlight the complex interplay of pro-inflammatory, angiogenic and regenerative factors within the exosomal cargo. The differential distribution of these biomolecules between exosomes and the broader secretome likely reflects distinct regulatory mechanisms governing inflammation and tissue repair, further emphasizing the functional specificity of exosome-mediated communication in wound healing. Moreover, exosomes provide added advantages in terms of storage, transport and safety. Future studies should focus on unraveling the mechanistic pathways underlying these shifts to further optimize exosome-based therapies for clinical applications. Papait et al. suggests that the active component for immune regulation resides in factors not conveyed in EVs, but in the whole secretome, which corroborates our findings [55].

The RT-PCR analysis revealed the amplification of several genes in exosomes derived from rHFSCs. The detection of p63, a well-established stemness marker, confirms the epithelial origin of these exosomes, as p63 plays a crucial role in maintaining the proliferative capacity of basal stem cells in stratified epithelia [43,56,57]. Additionally, the presence of CD34, a recognized bulge stem cell marker, suggests that the exosomes retain key stem-like properties of their parent HFSCs [46,58,59]. The expression of RUNX2 and IBSP, both key regulators of osteogenic differentiation, suggests that rHFSC-derived exosomes may contribute to osteogenesis or extracellular matrix remodeling. RUNX2 is a master transcription factor essential for bone formation, while IBSP encodes bone sialoprotein, a protein involved in biomineralization. The presence of these genes in exosomes implies that their molecular cargo could promote lineage-specific differentiation under appropriate microenvironmental cues [60,61,62]. Notably, IBSP was absent in the parent cells but present in exosomes. This suggests that exosomes may serve as a mechanism for intercellular communication, selectively transferring osteogenic signals even when the parent cells themselves do not express these genes. Such a process aligns with the idea that exosomes act as signaling vehicles, mediating differentiation and tissue remodeling responses. Interestingly, KRT15 was also detected in the exosomes despite being absent in the parent cells. KRT15 is typically found in quiescent HFSCs and is associated with self-renewal, epithelial homeostasis and regenerative capacity [63,64]. Its presence in exosomes suggests that rHFSC-derived exosomes may selectively package transcripts related to stemness maintenance and tissue repair, potentially influencing wound healing and epithelial regeneration in recipient cells. On the other hand, KRT14, KRT10 and KRT19, which are associated with differentiated keratinocyte lineages, were absent in both exosomes and parent cells [34,63,65,66]. This suggests that the exosomes predominantly contain transcripts linked to an undifferentiated state, rather than those associated with terminal differentiation. Additionally, the lack of COL2A1, ITGA6, ACAN, ITGB1, ADIPOQ and AAK1 indicates that rHFSC-derived exosomes do not strongly express markers of chondrogenic, adipogenic or mesenchymal differentiation, reinforcing their epithelial and stem-like profile [67,68,69]. The absence of KRT14, ACAN and KRT10 in both exosomes and parent cells suggest that these genes are not actively transcribed in the rHFSC population under the tested conditions. However, the presence of IBSP and KRT15 in exosomes—despite their absence in the parent cells—strongly suggests that exosomal RNA content does not simply reflect the cellular transcriptome but rather undergoes selective enrichment. This selective RNA packaging could be a regulatory mechanism where exosomes function to modulate recipient cell behavior by transferring specific transcripts. The enrichment of IBSP and KRT15 in exosomes, for instance, may indicate a role in promoting osteogenic and epithelial differentiation pathways through a paracrine signaling mechanism. These findings demonstrate that rHFSC-derived exosomes selectively encapsulate genetic material linked to stemness and differentiation, rather than passively reflecting the transcriptional profile of their parent cells. The differential expression of certain genes in exosomes compared to their originating cells suggests a targeted RNA sorting mechanism, potentially enhancing their role in tissue regeneration and repair. The presence of RUNX2, IBSP and KRT15 in exosomes highlights their potential in osteogenic and epithelial regeneration, whereas the absence of differentiation-associated keratins reinforces their stem-like nature [46,61,70,71,72,73,74]. Future studies should explore the functional impact of these exosomal transcripts on recipient cells in vitro and in vivo, to better understand their potential therapeutic applications in regenerative medicine.

SEM analysis confirmed the presence of exosomes, revealing their characteristic spherical morphology and nanoscale dimensions. The observed exosomal structures were consistent with previously reported size ranges, typically between 30 and 200 nm, further supporting their identification [75,76,77]. The uniformity in shape and size distribution suggests a well-defined exosome population, which is critical for ensuring reproducibility and therapeutic efficacy in biomedical applications. Additionally, the SEM images provided insights into the surface topology of the exosomes, indicating a smooth and intact membrane, which is essential for their stability and functionality in intercellular communication. The nanoscale features of the exosomes also suggest their suitability for efficient cellular uptake, an important factor in their role as carriers of bioactive molecules. These findings reinforce the potential of exosome-based therapies, as their structural integrity and size contribute to their ability to traverse biological barriers and deliver therapeutic cargo effectively. The EDS analysis provided further insight into exosome composition, detecting key elements such as carbon, oxygen, nitrogen, phosphorus and sulfur, indicative of lipids, proteins and nucleic acids. The presence of gold and palladium was linked to sample preparation, while trace elements like silicon, calcium, titanium and iron likely resulted from substrate interactions or from the Reaxon^TM^ tube used as support. Variations in elemental intensity across different regions suggest minor heterogeneity in surface composition or clustering.

Additionally, protein quantification was performed to determine the concentration of bioactive molecules, ensuring consistency across different batches [78,79]. The analysis confirmed that the total protein content remained within an acceptable range across samples, indicating a reproducible exosome production process. This consistency is crucial for maintaining the therapeutic potential of exosome-based treatments, as variations in protein concentration could impact their biological activity. Furthermore, the protein profile of exosomes was compared to that of the whole secretome, revealing key differences in the distribution of bioactive molecules, being the secretome higher in total protein content. The protein concentration in the plain secretome was higher than in the exosomes because the proteins were not only present within exosomes but also freely distributed in the secretome and associated with lipoproteins or extracellular complexes. While both contained important wound-healing factors, certain proteins were more enriched in either the exosome fraction or the broader secretome, suggesting differential packaging and secretion mechanisms. This comparison provided deeper insights into the functional properties of exosome preparations and their role in modulating wound healing processes. By ensuring uniform protein levels across different batches and understanding their relationship to the secretome, this analysis reinforces the reliability and therapeutic potential of exosome-based therapies for clinical applications.

To evaluate the wound-healing potential of rHFSC-derived exosomes and their secretome, in vitro functional assays were conducted using L929 fibroblast cells, a widely used model for studying wound repair due to their crucial role in connective tissue regeneration [80,81]. The PrestoBlue™ assay was used to assess cell viability and metabolic activity in response to exosome treatment, providing crucial data on their cytoprotective and proliferative effects. The results demonstrated that both exosomes and the secretome were cytocompatible with L929 cells, showing no cytotoxic effects. Furthermore, treatment with exosomes and secretome not only maintained cell viability but also significantly enhanced cell proliferation in the secretome group when compared to the control group. This increase in metabolic activity suggests that the bioactive molecules present in these preparations support cellular energy metabolism and promote cell growth, which is essential for effective wound healing. In addition, when evaluating the percentage of cell viability inhibition using the negative control group as a reference, both secretome and exosome treatments consistently demonstrated inhibition levels below the 30% threshold for cytotoxicity established by ISO 10993-5:2009 [49] across all timepoints. These results indicate that both the secretome and exosomes are cytocompatible with L929 cells.

The observed pro-proliferative effects may be attributed to key growth factors and cytokines, which are known to stimulate fibroblast activation, migration and ECM remodeling. The ability of exosomes and the secretome to enhance fibroblast viability and growth further highlights their regenerative potential, as fibroblasts play a key role in tissue repair by synthesizing collagen and other structural components necessary for wound closure. These findings reinforce the therapeutic relevance of exosome-based treatments, suggesting they could accelerate the wound-healing process by promoting cell survival and proliferation. Future studies should further explore the underlying molecular pathways and assess long-term effects on tissue regeneration.

The scratch assay, a classical in vitro wound healing model, was performed to examine cell migration and wound closure efficiency after treatment with rHFSC-derived exosomes and secretome, highlighting their ability to accelerate tissue repair [82,83]. As expected, in the absence of MMC, cells in the control group (DMEM + 10% FBS) exhibited a high migration capacity, leading to substantial wound closure over time. Treatment with exosomes and secretome further enhanced this process, suggesting their potential role in promoting fibroblast motility and tissue regeneration. Additionally, in the presence of MMC, wound closure was significantly impaired across all conditions, confirming that cell proliferation contributes to the healing process. However, even under these conditions, exosome- and secretome-treated groups showed slightly improved wound closure compared to the control, indicating a possible direct effect on cell migration independent of proliferation. These findings highlight the potential of exosomes and secretome in enhancing fibroblast migration and proliferation, key factors in wound healing, and suggest their therapeutic relevance for tissue regeneration applications.

The results of these analyses provided key insights into the regenerative properties of rHFSC-derived exosomes, further supporting their potential use in therapeutic applications.

Cooper et al. demonstrated that both the secretome and exosomes derived from human adipose stem cells improve cell migration and wound closure [84].

Villatoro et al. compared exosomes and secretome derived from canine bone marrow stem cells, canine adipose stem cells and feline adipose stem cells, finding that they exhibit comparable overall secretion profiles. However, bone marrow-derived stem cells produce higher levels of certain factors and exosomal content. These findings suggest that secretomes from all tested cell types are promising candidates for clinical applications in dogs. Importantly, the distinct characteristics of each cell source indicate that they may be better suited for different therapeutic purposes. Therefore, selecting the appropriate cell source should be based on the specific clinical application. Further studies investigating the functional differences between cell-derived products are needed to better guide clinicians in choosing the most effective cell product for targeted therapies [85,86].

## 4. Materials and Methods

### 4.1. rHFSCs-Derived Secretome and Exosomes Isolation

Conditioned medium 2D (CM2D) derived from rHFSCs was produced and extensively characterized for its wound healing potential, using previously established protocols as described in Sousa et al. [2].

After reaching a cell confluence of 70–80%, the culture medium was removed, and the flask was rinsed three times with DPBS, followed by two washes with DMEM-F12 medium (11039-021 Gibco^®^, Thermo Fisher Scientific, Waltham, MA, USA). Basal DMEM-F12 medium without antibiotics, antimycotics or Bovine Fetal Serum (FBS) was then added. The culture was incubated for 48 h under standard conditions. After incubation, the CM2D containing cell-secreted factors was collected, centrifuged and the secretome was stored at −20 °C until further use [2].

The rHFSC-derived exosomes were isolated using the total exosome isolation reagent from cell culture media (4478359 Invitrogen^®^, Thermo Fisher Scientific). The culture medium was harvested, centrifuged at 2000× *g* for 30 min to remove debris and cells and the supernatant was added to a new tube. The exosome isolation reagent was then added to the media, ensuring thorough mixing to promote exosome precipitation. Following an overnight incubation period at 4 °C to facilitate precipitation, the mixture was centrifuged at 10,000× *g* for 1 h at 4 °C. This step separated the exosomes, which formed a pellet, from the supernatant. The exosome pellet was then resuspended in DPBS and stored at −20 °C, until further use.

### 4.2. rHFSC-Derived Exosomes Analysis

Exosomes were isolated from passages P3 and P5 rHFSC-derived CM2D, stored at −20 °C and analyzed using multiplex LASER bead technology (Eve Technologies, Calgary, AB, Canada). The analysis targeted specific biomarkers using the Rat Cytokine/Chemokine 27-Plex Discovery Assay^®^ (RD27) and the TGFβ 3-Plex Discovery Assay^®^ Multi-Species Array (TGFβ1-3). Biomarkers examined included Epidermal Growth Factor Recombinant Protein (EGF), Granulocyte Colony-Stimulating Factor (G-CSF), Vascular Endothelial Growth Factor (VEGF), Interleukins: IL-6, IL-1α, IL-1β, IL-2, IL-4, IL-5, IL-10, IL-12p70, IL-13, IL-17A, IL-18, Regulated upon Activation, Normal T-Cell Expressed and Presumably Secreted (RANTES), Monocyte Chemoattractant Protein-1 (MCP-1), Tumor Necrosis Factor-Alpha (TNFα), Eotaxin, Fractalkine, Leptin, Interferon Gamma (IFNγ), Interferon-Gamma Inducible Protein (IP-10), Human Growth-Regulated Oncogene/Keratinocyte Chemoattractant/Cytokine-Induced Neutrophil Chemoattractant-1 (GRO/KC/CINC-1), Granulocyte-Macrophage Colony-Stimulating Factor (GM-CSF), LIX, Macrophage Inflammatory Proteins (MIP-1α, MIP-2) and Transforming Growth Factor Beta (TGFβ1, TGFβ2 and TGFβ3). Three independent samples were analyzed for each passage. Table 9 summarizes the biomarkers used and their role in wound healing.

### 4.3. Reverse Transcriptase Polymerase Chain Reaction (RT-PCR)

Exosomes derived from rHFSCs were used for the Polymerase Chain Reaction (PCR) analysis. Fifteen target genes, along with the two reference genes, beta-actin (ACTB) and glyceraldehyde 3-phosphate dehydrogenase (GAPDH) were amplified in separate reaction tubes. Total RNA was extracted from the exosomes using the TRIzol RNA extraction kit, following the manufacturer’s instructions, and cDNA was synthesized using reverse transcriptase.

The PCR reaction system consisted of SYBR green mix (10 µL), primer mix (1 µL), template (1 µL) and H_2_O (8 µL), forming a total reaction volume of 20 µL. It was loaded into Axygen PCR tubes, briefly centrifuged and then placed into the RT-PCR, using the SYBR green method. The thermocycling program included 40 cycles of 95 °C for 15 s, 60 °C for 15 s and 72 °C for 20 s. Each cDNA sample was processed in triplicate. The copy number for each cDNA sample was calculated based on a calibration curve generated by the PCR products for each gene.

The expression of 15 specific genes was analyzed to investigate molecular markers in exosomes derived from rHFSCs, focusing on their roles in key cellular differentiation pathways. Cell differentiation markers were examined, including osteogenic differentiation (RUNX2, IBSP), chondrogenic differentiation (COL2A1, ACAN) and adipogenic differentiation (ADIPOQ, AAK1), to assess the potential of exosome-mediated multilineage commitment. Furthermore, to assess the exosomal signature of rHFSCs, the study examined genes indicative of epithelial stem cell properties, including KRT19 and p63. CD34 was included as a marker for bulge stem cells. Additionally, KRT10 and KRT15 were analyzed to identify exosomal markers associated with the spinous and basal epithelial layers, and general keratinocytes, respectively.

The study also considered transmembrane or GPI-anchored proteins, such as ITGα6 and ITGβ1, known to be associated with the plasma membrane and/or endosomal compartments. Cytosolic proteins commonly found in EVs, including structural components like ACTB and metabolic enzymes such as GAPDH, were used for normalization of gene expression. Moreover, the study acknowledged the significant role of adhesion and ECM proteins, including COL2A1, in maintaining structural integrity and cell–matrix interactions [2,70].

For the gene expression analysis, a Prime PCR Custom Plate 96 Well from Bio Rad Laboratories^®^ (Hercules, CA, USA) was used, featuring 15 predesigned primers for the specified genes. This experimental setup allowed a detailed exploration of gene expression patterns in exosome-derived RNA, providing critical insights into the role of exosomes in cellular differentiation and characterization. Additionally, the analysis aimed to compare the gene expression profiles of exosomes with those of their parent rHFSCs to assess the extent of similarity and identify potential differences [2]. The inclusion of housekeeping genes for normalization ensured the accuracy and reliability of the obtained gene expression data, enabling a robust comparison between cellular and exosomal gene expression.

#### 4.3.1. RNA Isolation and cDNA Synthesis

Total RNA was isolated at room temperature following a phenol–chloroform extraction method combined with spin column purification. To each sample of exosomes, 200 μL of pre-warmed (37 °C) 2X Denaturing Solution was added and mixed thoroughly. An equal volume of Acid–Phenol/Chloroform was then added, followed by vigorous vortexing for 30–60 s. The samples were centrifuged at 10,000× *g* for 5 min at room temperature to separate aqueous and organic phases. The upper aqueous phase was carefully transferred to a fresh tube, and its volume was recorded. For RNA binding, 1.25 volumes of room-temperature 100% ethanol were added to the aqueous phase and mixed. The mixture was applied to a spin column and centrifuged at 10,000× *g* for 15 s, until all the sample passed through. The column was washed sequentially with 700 μL miRNA Wash Solution 1, followed by two washes with 500 μL Wash Solution 2/3, centrifuging after each wash. A final centrifugation at 10,000× *g* for 1 min ensured the removal of all residual wash buffers. RNA was eluted by applying 50 μL of preheated (95 °C) Elution Solution directly to the filter, followed by centrifugation. This elution step was repeated once, resulting in a total eluate volume of 100 μL. Isolated RNA was stored at ≤–20 °C until further use.

Prior to cDNA synthesis, RNA quantity and purity were evaluated using UV spectrophotometry on a nanodrop device (Implen GmbH, Isaza^®^, Munich, Germany). Purity was determined by measuring the A260/A280 ratio, which indicates protein contamination, and the A260/A230 ratio, which reflects the presence of polysaccharides, phenol or chaotropic salts. Acceptable purity thresholds were set between 2.0–2.2 for A260/A280 and 1.8–2.2 for A260/A230.

First-strand cDNA synthesis was performed using total RNA in a 20 µL reaction volume with the iScript™ cDNA Synthesis Kit (Bio-Rad Laboratories^®^, Hercules, CA, USA), following the manufacturer’s protocol. The reaction mixture was incubated in a T100™ Thermal Cycler (Bio-Rad Laboratories^®^) according to the specified kit conditions for time and temperature.

#### 4.3.2. Quantitative RT-PCR Assay

The RT-PCR assay was performed using the CFX Connect Real-Time PCR Detection System (BioRad Laboratories^®^). Standard PCR conditions were applied with iTaq™ Universal SYBR Green Supermix (BioRad Laboratories^®^), following the manufacturer’s guidelines. The system was used to analyze the expression of 15 target genes in exosomes derived from rHFSCs, with specific primer pairs designed for each gene. The temperature cycles recommended by the manufacturers were strictly followed.

After completing the RT-PCR, gene expression analysis was conducted. To ensure product specificity, melting curve analysis was performed. Threshold cycle (*Ct*) values of 39 were interpreted as indicative of weak reactions, which could suggest minimal presence of the target nucleic acid or potential environmental contamination. The ∆*Ct* value for each sample was calculated using the formula

$$\Delta Ct=Ct\left(target\;gene\right)-Ct\left(housekeeping\;gene\right)$$

This allowed for accurate normalization and reliable gene expression comparisons.

### 4.4. Scanning Electron Microscopy (SEM) and Energy Dispersive Spectroscopy (EDS)

A high-resolution Schottky Environmental Scanning Electron Microscope (FEI Quanta 400 FEG ESEM/EDAX Genesis X4M) was used for SEM and EDS analysis, equipped with X-ray Microanalysis and Electron Backscattered Diffraction (EBSD). The microscope operated in a high vacuum mode at an acceleration voltage of 15 kV. For sample preparation, 50 µL of exosomes isolated from cultured rHFSCs cells were fixed in 2% buffered glutaraldehyde (Merck^®^, G7651, Darmstadt, Germany) and deposited onto a Reaxon™ tube scaffold to facilitate sample handling and imaging. The fixed samples were then washed three times in 0.1M HEPES buffer (5 min cycles with gentle agitation). Dehydration was performed through a graded ethanol series (50%, 70%, 90% and 99%), with each concentration applied 2–3 times for 10–15 min. Subsequently, samples were infiltrated with a graded series of hexamethyldisilazane (HMDS – 440191, Merck^®^, Darmstadt, Germany) in ethanol for 15 min, followed by an additional 15 min incubation with pure HMDS. After HMDS removal, the plates were left to dry overnight in a laminar flow chamber to ensure complete evaporation. Prior to SEM and EDS analysis, samples were coated with a gold/palladium layer for 80 s using a 15 mA current to enhance conductivity and imaging quality.

### 4.5. Total Protein Quantification

Total protein content was measured using the Pierce™ Dilution-Free™ Rapid Gold BCA Protein Assay (A55860, Thermo Scientific™, Waltham, MA, USA) and by measuring absorbance at 280 nm using a NanoDrop spectrophotometer (NanoDrop Technologies, Wilmington, DE, USA). The Pierce™ Dilution-Free™ Rapid Gold BCA Protein Assay was used to determine the protein concentration of samples following the manufacturer’s instructions. Briefly, 10 µL of each sample or standard was directly added to 200 µL of the working reagent in a 96-well plate. The plate was incubated for 5 min, allowing the bicinchoninic acid (BCA) to react with protein-bound cuprous ions in an alkaline medium, forming a purple–gold complex. Absorbance was measured at 450 nm using a microplate reader, with a secondary measurement at 570 nm for background correction. Protein concentrations were calculated based on a BSA standard curve prepared in parallel. All samples and standards were analyzed in triplicate to ensure accuracy and reproducibility.

### 4.6. Prestoblue Assay

To determine the cytocompatibility of the secretome and exosomes derived from the rHFSCs, the PrestoBlue™ viability assay was performed. L929 cells were seeded at 8000 cells/cm^2^ in a 24-well plate and were incubated overnight at 37 °C in a humidified atmosphere (80%) with 5% CO_2_. At specific timepoints (24, 72 and 168 h), fresh complete medium containing 10% (*v*/*v*) Presto Blue™ reagent was added and incubated for 1 h at 37 °C in a 5% CO_2_, 80% humidified atmosphere. Afterwards, the supernatant was collected and transferred to a 96-well plate for absorbance readings at 570 nm and 595 nm. The wells were then washed with DPBS to remove Presto Blue™ residues, and fresh culture medium was added.

The study included two experimental groups: rHFSCs-derived secretome and rHFSCs-derived exosomes (100 µL + DMEM 10%), as well as the negative (DMEM 10%) and positive control groups (DMEM 10% + Dimethyl Sulfoxide (DMSO 10%). The normalized value for each well was calculated by subtracting the absorbance at 595 nm from that at 570 nm.

Absorbance measurements were performed in triplicate using the Multiskan™ FC Microplate Photometer (Thermo Scientific™, 51119000, Thermo Fisher Scientific, Waltham, MA, USA). Data were expressed as a percentage of viability inhibition relative to the control group.

### 4.7. Scratch Assay

L929 fibroblasts were seeded at a density of 8000 cells/cm^2^ in 6-well plates and cultured at 37 °C in a 5% CO_2_ atmosphere until they reached ≥90% confluence. One group was pre-treated with mitomycin C (MMC) for 2 h to inhibit DNA synthesis, allowing differentiation between cell migration and proliferation during the regeneration process. After the incubation period, a sterile 200 μL micropipette tip was used to scrape the cell monolayer, creating a uniform scratch. Detached cells and debris were removed by washing twice with DPBS. Cells were then incubated with either culture medium containing 100 μL of exosomes derived from rHFSCs, 100 μL of secretome from rHFSCs or neither (Control) and in triplicates. Cell migration into the scratch area was monitored at 0, 2, 4, 6, 8, 10, 12, 24, 32 and 53 h using an EVOS M5000 microscope. Quantitative analysis of cell movement was performed using ImageJ software version 1.54d (NIH, Bethesda, MD, USA). The wound closure percentage was calculated using the formula

$$Wound\;closure=100\times \frac{\left(Initial\;Area-Final\;Area\right)}{Initial\;Area}$$

### 4.8. Statistical Analysis

The statistical analysis was conducted using GraphPad Prism version 8.00 for Windows (GraphPad Software 8, La Jolla, CA, USA). Data, when appropriate, were presented as mean ± standard error of the mean (SEM). The normality of the data was assessed using the Shapiro–Wilk test. For comparisons between two groups, unpaired t-tests were used, while differences involving multiple groups or factors were evaluated using two-way ANOVA followed, when appropriate, by Tukey’s multiple comparisons post-hoc test. A significance threshold of <0.05 was considered statistically significant. The significance of the results is indicated by the symbols (*), with (*) corresponding to 0.01 ≤ *p* < 0.05, (**) to 0.001 ≤ *p* < 0.01, (***) to 0.0001 ≤ *p* < 0.001 and (****) to *p* < 0.0001.

## 5. Conclusions

This study presents a comprehensive analysis of exosomes and secretome derived from rHFSCs, highlighting their therapeutic potential in skin regeneration. Through detailed characterization and in vitro functional assays, the research demonstrates that these extracellular components contain key bioactive molecules involved in cell migration, proliferation and extracellular matrix remodeling, contributing to accelerated wound closure. The findings support the relevance of both exosome- and secretome-based approaches for regenerative medicine, while also emphasizing the need for further in vivo validation, standardization and scalability. Despite limitations such as donor variability, reliance on in vitro models and species-specific differences, this work provides important insights and establishes a strong foundation for the development of rHFSC-derived exosomes as a promising, cell-free therapeutic strategy. Future studies will focus on evaluating their efficacy and mechanisms of action in animal wound healing models to advance their translational potential.

## Figures and Tables

**Table 1:** rHFSC-derived exosomes and secretome analysis with mean concentration values for each biomolecule in P3 and P5 (mean ± SEM).

Biomolecule	Exosomes Mean ± SEM (P3)	Exosomes Mean ± SEM (P5)	Secretome Mean ± SEM (P3)	Secretome Mean ± SEM (P5)
EGF	0.10 ± 0.01	0.1 ± 0.02	0.16 ± 0.03	0.18 ± 0.04
Eotaxin	1.79 ± 0.14	1.65 ± 0.00	0.16 ± 0.00	0.00 ± 0.00
Fractalkine	1.17 ± 0.19	1.47 ± 0.54	4.38 ± 0.29	3.06 ± 0.15
GM-CSF	20.10 ± 3.95	28.06 ± 4.02	35.39 ± 10.69	39.75 ± 11.55
GRO/KC/CINC-1	90.97 ± 8.13	85.26 ± 8.73	47.32 ± 8.64	57.64 ± 7.24
IFNγ	15.31 ± 1.68	12.31 ± 2.20	34.01 ± 2.26	30.67 ± 0.73
IL-1α	5.85 ± 0.61	7.97 ± 3.67	3.79 ± 2.27	12.83 ± 5.50
IL-1β	4.42 ± 0.83	4.29 ± 0.23	6.99 ± 0.86	7.27 ± 0.63
IL-2	4.40 ± 0.71	7.24 ± 1.23	12.21 ± 1.23	12.65 ± 0.58
IL-4	3.71 ± 0.68	2.54 ± 1.17	4.88 ± 1.30	2.63 ± 0.00
IL-5	6.50 ± 1.42	8.68 ± 1.32	24.13 ± 2.69	21.13 ± 2.46
IL-6	104.20 ± 30.96	119.66 ± 21.91	166.36 ± 50.22	165.62 ± 0.00
IL-10	6.24 ± 1.87	6.42 ± 0.83	18.37 ± 1.87	18.17 ± 0.88
IL-12p70	8.80 ± 0.68	6.08 ± 2.04	4.34 ± 1.80	1.42 ± 0.00
IL-13	4.43 ± 0.45	3.54 ± 1.34	3.39 ± 0.43	8.84 ± 0.51
IL-17A	2.94 ± 0.63	1.77 ± 0.58	4.77 ± 0.30	7.57 ± 1.11
IL-18	3.68 ± 0.63	3.05 ± 0.00	8.89 ± 1.31	5.55 ± 1.24
IP-10	0.61 ± 0.1	0.79 ± 0.21	2.33 ± 0.31	1.96 ± 0.38
Leptin	35.04 ± 7.41	32.97 ± 12.35	32.31 ± 8.97	30.49 ± 4.08
LIX	6.29 ± 0.00	0.00 ± 0.00	0.00 ± 0.00	0.00 ± 0.00
MCP-1	77.34 ± 28.53	37.68 ± 19.27	131.64 ± 38.26	137.21 ± 19.77
MIP-1α	0.40 ± 0.20	0.40 ± 0.34	1.70 ± 0.57	2.19 ± 0.39
MIP-2	13.81 ± 0.00	31.83 ± 0.00	32.78 ± 5.77	44.02 ± 7.97
RANTES	0.23 ± 0.01	0.24 ± 0.00	0.51 ± 0.03	0.50 ± 0.01
TNFα	0.17 ± 0.03	0.13 ± 0.07	0.00 ± 0.00	0.00 ± 0.00
VEGF	14.77 ± 2.43	19.47 ± 1.60	91.75 ± 3.22	98.88 ± 1.06
G-CSF	0.00 ± 0.00	0.00 ± 0.00	0.00 ± 0.00	0.00 ± 0.00
TGF-β1	0.00 ± 0.00	0.00 ± 0.00	5.82 ± 1.07	7.14 ± 1.02
TGF-β2	0.99 ± 0.07	0.90 ± 0.00	0.97 ± 0.04	0.98 ± 0.00
TGF-β3	0.00 ± 0.00	0.00 ± 0.00	0.19 ± 0.00	0.00 ± 0.00

**Table 2:** Statistical differences identified between groups.

	Exosomes P3 vs. Exosomes P5	Exosomes P3 vs. Secretome P3	Exosomes P3 vs. Secretome P5	Exosomes P5 vs. Secretome P3	Exosomes P5 vs. Secretome P5	Secretome P3 vs. Secretome P5
IL-1α	ns	*	ns	***	ns	****
IL-2	ns	*	**	ns	ns	ns
IL-5	ns	****	****	****	****	ns
IL-10	ns	****	****	****	****	ns
IL-12p70	**	****	****	ns	***	ns
IL-13	ns	ns	ns	ns	ns	**
IL-17a	ns	ns	ns	ns	*	ns
LIX	**	**	ns	ns	***	**
TGF-β1	ns	ns	*	ns	*	ns
MCP-1	*	ns	ns	**	**	ns
VEGF	ns	*	*	ns	*	ns
GRO/KC/CINC-1	ns	***	**	*	ns	ns

Results significances are presented through the symbol (*), according to the *p*-value, (*) corresponding to 0.01 ≤ *p* < 0.05, (**) to 0.001 ≤ *p* < 0.01, (***) to 0.0001 ≤ *p* < 0.001 and (****) to *p* < 0.0001. (ns = no statistically significant differences).

**Table 3:** Average Ct and ΔCt values for genes under study.

Target Gene	Ct Average	ΔCt
*KRT14*	nd	nd
*p63*	4.92 ± 0.00	−31.1
*CD34*	33.40 ± 1.41	−2.6
*COL2A1*	nd	nd
*ITG* *α6*	nd	nd
*ACAN*	nd	nd
*ITG* *β1*	Nd	nd
*RUNX2*	19.45 ± 0.00	−16.5
*KRT10*	nd	nd
*IBSP*	39.67 ± 0.00	3.7
*KRT15*	39.19 ± 0.00	3.2
*ADIPOQ*	nd	nd
*AAK1*	nd	nd
*KRT19*	nd	nd

nd = non-defined.

**Table 4:** Statistical differences identified between groups.

	Secretome P3	Secretome P5	Exosomes P3	Exosomes P5
Secretome P3		ns	*	ns
Secretome P5			ns	*
Exosomes P3				ns

Results significances are presented through the symbol (*), according to the *p*-value, (*) corresponding to 0.01 ≤ *p* < 0.05. (ns = no statistically significant differences).

**Table 5:** Statistical differences identified between groups.

	24 h				72 h				168 h			
	Exosomes	Secretome	DMEM 10%	DMSO 10%	Exosomes	Secretome	DMEM 10%	DMSO 10%	Exosomes	Secretome	DMEM 10%	DMSO 10%
Exosomes		*	****	****		****	****	****		****	****	****
Secretome			****	****			ns	****			****	****
DMEM 10%				****				****				****

Results significances are presented through the symbol (*), according to the *p*-value, with (*) corresponding to 0.01 ≤ *p* < 0.05 and (****) to *p* < 0.0001. (ns = no statistically significant differences).

**Table 6:** Statistical differences identified between groups.

	24 h		72 h		168 h	
	Secretome	DMSO 10%	Secretome	DMSO 10%	Secretome	DMSO 10%
Exosomes	ns	****	**	****	****	**
Secretome		***		****		***

Results significances are presented through the symbol (*), according to the *p*-value, with (**) to 0.001 ≤ *p* < 0.01, (***) to 0.0001 ≤ *p* < 0.001 and (****) to *p* < 0.0001. (ns = no statistically significant differences).

**Table 7:** Statistical differences identified between groups.

No MMC									
	2 h	4 h	6 h	8 h	10 h	12 h	24 h	32 h	53 h
Control vs. Exosomes	ns	ns	ns	ns	ns	*	ns	ns	ns
Control vs. Secretome	ns	**	*	*	ns	*	ns	ns	ns
Exosomes vs. Secretome	ns	ns	ns	ns	ns	ns	ns	ns	ns

Results significances are presented through the symbol (*), according to the *p*-value, with (*) corresponding to 0.01 ≤ *p* < 0.05 and (**) to 0.001 ≤ *p* < 0.01. (ns = no statistically significant differences).

**Table 8:** Statistical differences identified between groups.

With MMC									
	2 h	4 h	6 h	8 h	10 h	12 h	24 h	32 h	53 h
Control vs. Exosomes	ns	ns	*	ns	ns	ns	ns	ns	ns
Control vs. Secretome	ns	ns	ns	ns	ns	**	ns	ns	ns
Exosomes vs. Secretome	ns	ns	ns	ns	ns	ns	ns	ns	ns

Results significances are presented through the symbol (*), according to the *p*-value, with (*) corresponding to 0.01 ≤ *p* < 0.05 and (**) to 0.001 ≤ *p* < 0.01. (ns = no statistically significant differences).

**Table 9:** Biomarkers and their wound healing role.

Biomarker	Function in Wound Healing
EGF	Promotes keratinocyte and fibroblast proliferation, aiding re-epithelialization and collagen synthesis [87,88].
G-CSF	Enhances neutrophil production, supporting debris clearance during the inflammatory phase [89,90].
VEGF	Critical for angiogenesis, ensuring oxygen and nutrient delivery to healing tissues [91,92].
IL-6, IL-1α and IL-1β	Key pro-inflammatory cytokines that regulate inflammation, recruit immune cells and stimulate fibroblasts and keratinocytes [50].
IL-2 and IL-12p70	Primarily modulate immune responses, indirectly affecting wound healing [93,94].
IL-4 and IL-13	Promote fibroblast differentiation into myofibroblasts, impacting wound contraction and fibrosis [95,96].
IL-5 and Eotaxin	Mainly recruit eosinophils, with limited direct impact on typical wound healing [97].
IL-10	Anti-inflammatory cytokine, crucial for resolving inflammation and minimizing scarring [98,99].
IL-17A and IL-18	Contribute to inflammation and influence keratinocyte activity and angiogenesis [100,101].
RANTES (CCL5), MCP-1 (CCL2), MIP-1α (CCL3) and MIP-2 (CXCL2)	Chemokines that recruit immune cells to the wound site, supporting inflammation and repair [102,103].
TNFα	It stimulates the production of other cytokines and chemokines, activates immune cells and can influence fibroblast and keratinocyte behavior. Drives early inflammation but may impair healing if chronically elevated [104,105].
Fractalkine (CX3CL1)	Aids immune cell recruitment and endothelial interaction [106,107].
Leptin	Supports keratinocyte proliferation, angiogenesis and collagen production [108,109].
IFNγ and IP-10 (CXCL10)	Influence inflammation and ECM remodeling, with prolonged expression potentially impairing healing [110,111].
GRO/KC/CINC-1 (CXCL1) and LIX (CXCL5)	Attract neutrophils during early wound responses [112].
GM-CSF	Promotes differentiation of immune cells, supporting both inflammation and repair [113].
TGFβ1 and TGFβ2	Stimulate fibroblast proliferation, myofibroblast differentiation and ECM production [114].
TGFβ3	Encourages regenerative healing with reduced scarring [115].

## Data Availability

Further data on the reported results are available from the corresponding author on request.

## Abbreviations

The following abbreviations are used in this manuscript:

ACTB	Beta-Actin
Au	Gold
BCA	Bicinchoninic Acid
C	Carbon
Ca	Calcium
CM2D	Conditioned Medium 2D
Ct	Threshold cycle
DMSO	Dimethyl Sulfoxide
EBSD	Electron Backscattered Diffraction
ECM	Extracellular Matrix
EDS	Energy-Dispersive X-ray Spectroscopy
EGF	Epidermal Growth Factor Recombinant Protein
EVs	Extracellular Vesicles
FBS	Bovine Fetal Serum
Fe	Iron
GAPDH	Glyceraldehyde 3-phosphate Dehydrogenase
G-CSF	Granulocyte Colony-Stimulating Factor
GM-CSF	Granulocyte-Macrophage Colony-Stimulating Factor
GRO/KC/CINC-1	Human Growth-Regulated Oncogene/Keratinocyte Chemoattractant/Cytokine-Induced Neutrophil Chemoattractant-1
HMDS	Hexamethyldisilazane
IFNγ	Interferon Gamma
IL	Interleukin
IP-10	Interferon-Gamma Inducible Protein
MCP-1	Monocyte Chemoattractant Protein-1
MIP	Macrophage Inflammatory Protein
MMC	Mitomycin C
MSCs	Mesenchymal Stem Cells
N	Nitrogen
O	Oxygen
P	Phosphorus
PCR	Polymerase Chain Reaction
Pd	Palladium
RANTES	Regulated upon Activation, Normal T-Cell Expressed and Presumably Secreted
rHFSCs	Rat Hair Follicle Stem Cells
RT-PCR	Reverse Transcriptase Polymerase Chain Reaction
S	Sulfur
SEM	Standard Error of the Mean
Si	Silicon
TGFβ	Transforming Growth Factor Beta
Ti	Titanium
TNFα	Tumor Necrosis Factor-Alpha
UP	University of Porto
VEGF	Vascular Endothelial Growth Factor
Z	Zone
